# Guide to the structural characterization of protein aggregates and amyloid fibrils by CD spectroscopy

**DOI:** 10.1002/pro.70066

**Published:** 2025-02-19

**Authors:** József Kardos, Márton Péter Nyiri, Éva Moussong, Frank Wien, Tamás Molnár, Nikoletta Murvai, Vilmos Tóth, Henrietta Vadászi, Judit Kun, Frédéric Jamme, András Micsonai

**Affiliations:** ^1^ Department of Biochemistry, Institute of Biology ELTE Eötvös Loránd University Budapest Hungary; ^2^ ELTE NAP Neuroimmunology Research Group, Department of Biochemistry, Institute of Biology ELTE Eötvös Loránd University Budapest Hungary; ^3^ ELTE—Functional Nucleic Acid Motifs Research Group, Department of Biochemistry, Institute of Biology ELTE Eötvös Loránd University Budapest Hungary; ^4^ DISCO Beamline Synchrotron SOLEIL Gif‐sur‐Yvette France

**Keywords:** Alzheimer's disease, amyloid formation, amyloid‐β, BeStSel, circular dichroism spectroscopy, protein aggregation, secondary structure estimation

## Abstract

Protein aggregation and amyloid formation are linked to numerous degenerative diseases, such as Alzheimer's or Parkinson's disease. Additionally, protein aggregation plays a crucial role in various biological processes, such as storage of molecules or cell signaling. Protein molecules can form a wide range of aggregates, from oligomers of different sizes to non‐specific aggregates and highly ordered cross‐β structured amyloid fibrils with diverse morphologies. Circular dichroism (CD) spectroscopy is a widely used technique to study protein structures providing detailed information at the secondary structure level, and is ideal to distinguish and characterize protein aggregates. Despite its potential, CD spectroscopy is often perceived as having limited application on protein aggregates due to challenges, such as sample inhomogeneity, precipitation, light scattering and other factors that complicate accurate analysis. In this study, we present a detailed protocol for examining the structure of protein aggregates and amyloid fibrils using CD spectroscopy. We outline the optimal experimental conditions for sample preparation and demonstrate how to identify and mitigate various interfering effects, using specific examples of disease‐related amyloidogenic proteins. We also discuss the instrumental parameters, baseline subtraction, normalization, and quality control of CD spectra. Furthermore, we evaluate the performance of different secondary structure estimating algorithms on amyloid fibril CD spectra highlighting the superiority of BeStSel and CDNN. Our findings could enhance the structural analysis of protein aggregates, contributing to a better understanding of associated diseases and the development of new therapeutic strategies.

## 1 INTRODUCTION

Circular dichroism (CD) spectroscopy has become a widely used and standard technique in protein science to study the structure of protein molecules (Greenfield, 2006; Kelly et al., 2005). The far‐UV CD spectrum, mostly originated from the electronic transitions of the peptide groups (n → π* and π → π* transitions) in the asymmetrical environment of the protein molecule, is highly sensitive and characteristic for the conformation of the peptide backbone (Woody, 1995). Although it is a low‐resolution technique, providing structural information mainly at the protein secondary structure level, its advantages are the fast/high throughput measurement capability, the wide usable protein concentration range, the variability of the environmental conditions (temperature, solvent, buffer composition, salt concentration, pH, the presence of additives, or even dry films are measurable), the simple data analysis with available tools, its low cost and robust, reliable instrumentation. It is often applied as a complementary technique of high resolution methods. Protein CD spectroscopy is widely used in all fields of protein science, from basic studies of protein structure to biotechnology and pharmaceutical industry, addressing protein structure, stability and interactions (Fasman, 1996; Greenfield, 2006; Jones, 2022). The near‐UV CD spectrum of the proteins in the 250–300 nm wavelength range mainly reflects the environment of the aromatic side‐chains and disulfide bonds. It cannot be directly used for structure determination, however, it is a fingerprint and sensitive for any structural changes. Thus, its change upon aggregation and amyloid formation is indicative of the contribution of the corresponding aromatic residues in the process. Moreover, the loss of the signal in the near‐UV is an indication of disorder, if an aromatic side‐chain is in disordered structure and fully accessible for the solvent, its environment averages out and the CD signal disappears.

Protein aggregation and amyloid formation of various peptides and proteins are associated with degenerative diseases including Alzheimer's, Parkinson's, Huntington's disease, and dialysis related amyloidosis (Scarpioni et al., 2016; Stefani & Dobson, 2003). Depending on the disease, the protein molecule can form different types of aggregates, such as oligomers, non‐specific aggregates, and amyloid fibrils of various morphologies and structures. These aggregates can form intra‐ or extracellularly, might be toxic and can deposit in the living tissues causing serious complications. An extreme example of protein aggregation is the transmissible prion disease where the highly stable scrapie form of prion protein, as template, can convert the native protein to the toxic structure with high efficiency (Jeffrey et al., 1995). However, there exist functional, healthy amyloids from bacteria to mammals, having diverse roles from biofilm formation to storage of peptide hormones (Otzen & Riek, 2019). To understand the (patho)physiological effects, distinguish the different forms of protein aggregates and amyloid fibrils and interfere with the aggregation process, it is indispensable to have suitable structure determination tools. However, the investigation of protein aggregates is complicated because they are often inhomogeneous, might be insoluble, non‐crystallizable, and they can be too large for solution NMR. For high resolution studies of amyloid fibrils, X‐ray crystallography and solid‐state NMR have been used (Daskalov et al., 2021; Eisenberg & Sawaya, 2017; Liu et al., 2024), and recently, cryo‐EM has become an emerging technique with increasing structure deposits in the PDB (Chua et al., 2022; Fitzpatrick & Saibil, 2019; Scheres et al., 2023). However, the structures of amyloid fibrils represent only a small fraction of the PDB; around 720 structures of 56 proteins (15% X‐ray, 8% NMR, and 73% cryo‐EM) out of 225,000 protein structures were deposited by the end of 2024 indicating the highly challenging task of amyloid structure determination. At the level of secondary structure, CD spectroscopy, with all the above mentioned advantages, is a favorable and popular tool to study the structure, kinetics, stability and the effect of additives/inhibitor molecules in protein aggregation and amyloid fibril formation. It is a standard technique in basic studies on the mechanism of aggregation and amyloid formation of disease related proteins such as amyloid‐β peptide, α‐synuclein, transthyretin, huntingtin, β_2_‐microglobulin, lysozyme, or insulin (Abelein et al., 2013; Adachi et al., 2015; Babenko et al., 2011; Breydo et al., 2012; Buell, 2022; Bulyaki et al., 2021; Ghodke et al., 2012; Ikenoue et al., 2014; Muta et al., 2014; Nitani et al., 2017; Serpell et al., 2000; So et al., 2017; Zhang et al., 2019) and mostly used to show the conformational change and β‐sheet formation of the protein upon aggregation. However, to gather high quality, quantitative and usable results on such problematic samples, special attention should be paid to sample preparation, experimental settings and data analysis. It is common, that the results are of unusable quality or allow only qualitative interpretations or comparisons, which, unfortunately, is not always recognized by researchers and reviewers and we can see articles in respected journals with misinterpreted poor quality data. Moreover, the secondary structure analysis of the CD spectra might be carried out by using non‐suitable algorithms, which cannot handle the unique β‐sheet structures of amyloid fibrils.

The aim of the present work is to provide a detailed protocol for the structural studies of protein aggregates and amyloid fibrils by CD spectroscopy to researchers not fully familiar with all the requirements needed for proper sample preparation, data collection and analysis, secondary structure estimation, and critical interpretation of the results. We will explain all the possible issues, their manifestation in the CD spectra and make recommendations how to avoid them or decrease their contribution. There is a great demand for this work, because the detailed protocols published so far are general protocols on protein CD spectroscopy, without discussing the specifics of investigating protein aggregates and amyloid fibrils, these are rather mentioned as sample problems (Greenfield, 2006; Kelly et al., 2005). Some protocols on the methods to study of amyloid fibrils present CD spectroscopy (Vadukul et al., 2019), however, providing only a brief technical description with no quantitative structural analysis. We have to note that here we do not deal with the study of large inhomogeneous protein aggregates, such as photosynthetic complexes or large filamentous structures such as actin filaments or microtubules that exhibit psi‐type CD spectra (Kim et al., 1986).

## 2 RESULTS

### 2.1 Overview of the protocol for the study of protein aggregates by CD spectroscopy

**FIGURE 1:**
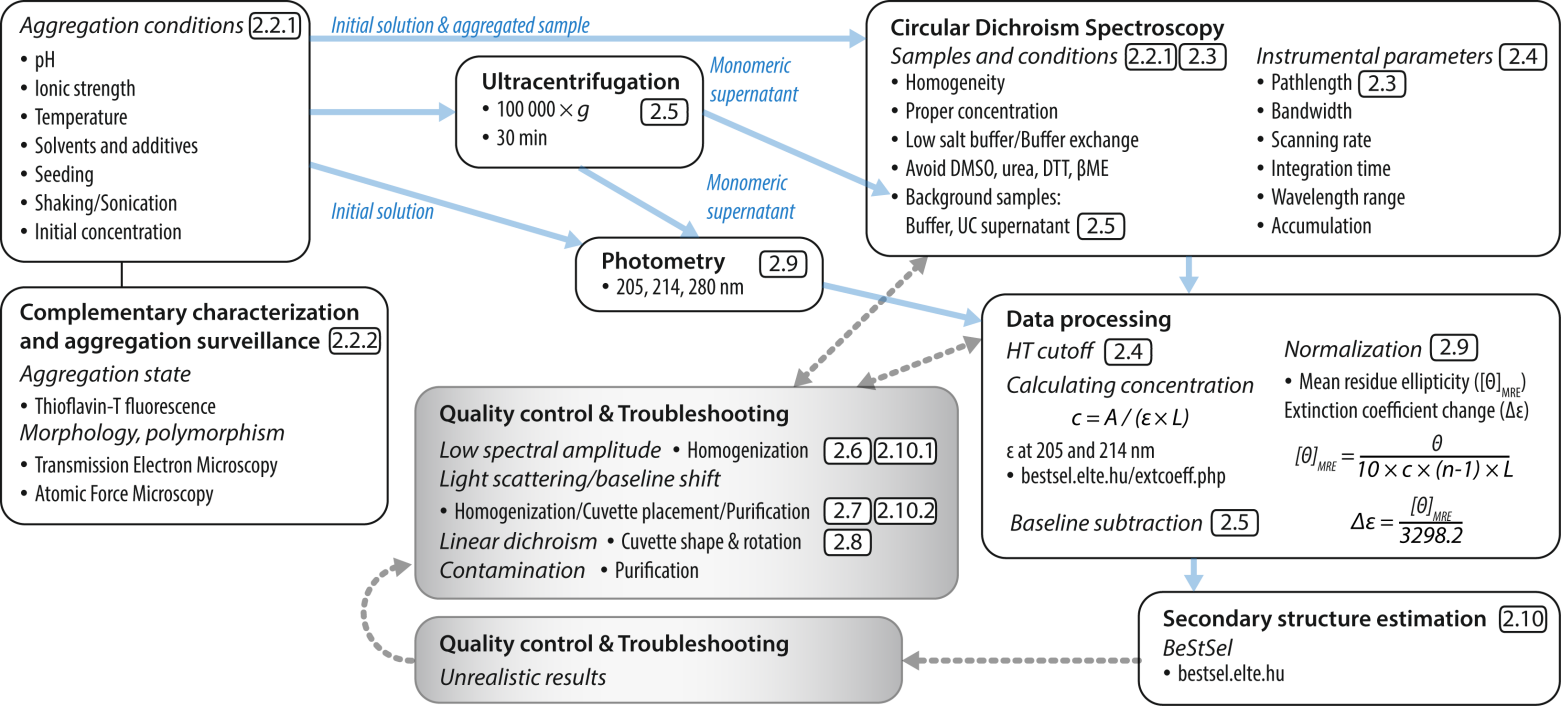
Schematic workflow of secondary structure investigation of protein aggregates by CD. The related sections of the manuscript for detailed descriptions are indicated.

Our protocol for the structural analysis of protein aggregates by CD (Figure 1) starts with the main aspects of sample preparation. Not all the aggregated protein/amyloid samples can be measured by CD. The sample properties need to meet several criteria from the side of the protein aggregates and the side of the buffer composition resulting a sample with suitable optical properties for CD spectroscopy free from distorting effects and artifacts. Characterization of the samples with complementary techniques such as thioflavin‐T fluorescence to show the presence of amyloid fibrils and imaging with TEM or AFM for aggregate morphology, size and composition are useful for the interpretation of the structural results from CD. For CD, the sample should be well homogenized, free of visible precipitates and its overall absorbance should be below a limit to keep the detector voltage in the optimal range which requires careful choice of the protein concentration, buffer composition and pathlength. We need to recognize and minimize the disturbing effects that interfere with CD spectrum collection and structural analysis, such as inhomogeneity, differential light scattering, and linear dichroism. The recording of the CD spectra of aggregated protein samples requires the same instrument parameter setup as measuring other protein solutions, however, due to the often extreme sample properties (high absorption, noisier data), light intensity (proportional to bandwidth) and data collection time should be maximized. The proper baseline subtraction is essential. To produce the spectrum of the aggregates/amyloid fibrils, free of the monomer contribution, the preferred baseline is the spectrum of the supernatant of ultracentrifuged sample, containing only the monomer protein fraction. The accurate concentration determination of the overall sample and the supernatant is crucial for the correct normalization of the spectra. Most often, concentration is determined by photometry using the proper extinction coefficients calculated from the primary sequences. The measured, baseline‐subtracted and normalized CD spectrum should be inspected for the signs of artifacts or disturbing contributions which might be indicated by distorted spectral shape, decreased or unexpected abnormal amplitudes. In some cases, the collected CD spectra can only be used for qualitative comparisons, which might still be important and useful to distinguish different aggregates and amyloid fibrils. After quality control, CD spectra can be analyzed for secondary structure composition by using one of the available algorithms. Comparative studies show that the BeStSel method is the most appropriate for this purpose being capable of distinguishing different types of β‐structures, the main structural components of protein aggregates, with special attention to parallel β‐sheets, which is usually characteristic of amyloid fibrils (Micsonai et al., 2015, 2018). Studies have also been carried out to characterize early stage amyloid structures mainly using NMR and CD spectroscopy for α‐synuclein (Skamris et al., 2019), amyloid‐β (Santoro et al., 2021), hIAPP (Guo et al., 2018), and using fluorescence‐based techniques for several amyloidogenic proteins (Li et al., 2024). We have to note that BeStSel overperforms other existing CD spectrum analysis methods in any type of secondary structures (Micsonai et al., 2015, 2018, 2022), thus it can be universally applied. Reasonable results of BeStSel provide the secondary structure composition of the protein aggregates/amyloid fibrils and can be basis of further studies, interpretation of aggregation mechanism, model building, development of inhibitors, etc.

#### 2.1.1 Sample preparation—experimental conditions for CD

Depending on the aggregation propensity and physico‐chemical properties of the protein or peptide, aggregation can occur under a wide variety of solution conditions from physiological conditions to extreme conditions and typically can be induced by low pH, high ionic strength, high temperature, presence of co‐solvents (such as fluorinated alcohols), in the presence of moderate concentrations of detergents, chemical denaturants or other additives (Abelein et al., 2013; Adachi et al., 2015; Babenko et al., 2011; Buell, 2022; Bulyaki et al., 2021; Ghodke et al., 2012; Kardos et al., 2011; Lin et al., 2016; Muta et al., 2014; Noji et al., 2021; So et al., 2017; Zhang et al., 2019). A physical agitation, shaking or sonication of the sample solution usually facilitates the formation of aggregates (So et al., 2011). The aggregation process is highly sensitive to the protein concentration, as well. Depending on the protein and the experimental conditions, an aggregate‐monomer thermodynamic equilibrium might be reached, which is determined by the stability of the aggregated form relative to the monomers. Aggregates of various morphology can be formed from small oligomers to non‐specific aggregates and amyloid fibrils with highly ordered β‐sheet structures, and often the mixtures of these co‐exist in the sample. The growth of amyloid fibrils is usually a nucleation dependent process with a lag‐phase, which can be excluded by inoculating the monomer solution with preformed fibril seeds. Such template seeds can be isolated from biopsy of diseased tissue and might preserve the morphology of the amyloid (or prion) strain upon extension with recombinant protein in vitro and thus be an important target for structural studies. We do not provide a detailed protocol here on the preparation of protein aggregates and amyloid fibrils, only discuss important aspects of their sample preparation and buffer conditions in relation to the CD measurements. For CD, there is a wide range of suitable buffer conditions. The most important aspects are the favorably low absorbance of the buffer components in the measured wavelength range to keep the photomultiplier voltage below the limit (see in Section 2.3) and their achiral nature. The buffer and salt concentrations should be kept as low as possible. For thorough list of absorptions and wavelength limits of various compounds used in samples for CD spectroscopy, please refer to Micsonai et al. (2021). Generally, 10–20 mM buffer and 100 mM NaCl concentration allows data collection in a 1 mm cuvette in the 200–260 nm, narrowest necessary wavelength range. Before preparing the protein solutions, it is recommended to carry out a test measurement with the buffer solution to be used. When higher salt concentration is needed or we aim to collect data in a wider wavelength range, a cuvette with shorter pathlength (with higher protein concentrations) should be used. Alcohols are usually compatible with CD experiments. DMSO, which is often used for solubilizing or monomerizing peptides, should be completely avoided because of its high absorbance even at low concentration. Chemical denaturants, such as urea or GdnHCl at the normally used high concentrations do not allow data collection in the wavelength ranges required for secondary structure analysis. Reducing agents, dithiothreitol, and β‐mercaptoethanol should be avoided (at least kept below 0.5 mM), TCEP is their preferred substitute.

In case the growth conditions for the aggregates are not directly compatible with CD measurements, the solutions might be diluted in an appropriate buffer or can be transferred to an appropriate buffer with a concentrator tube, dialysis or centrifugation and re‐suspension. If the protein concentration allows (see Section 2.3), a cell with a short pathlength might solve the problem.

The aggregate sample suitable for CD measurement might be viscous but should be transparent, without large precipitates or pellet. Highly opalizing or precipitated solutions cannot be used for CD. We have to note that aggregated protein samples should not be frozen, freeze and thaw induces the formation of large precipitates difficult to homogenize which interfere with the CD measurements. In the case of strong precipitation, infrared (FTIR) spectroscopy might be a complementary technique, which is capable of distinguishing β‐sheet structures (Ruysschaert & Raussens, 2018; Sarroukh et al., 2013).

#### 2.1.2 Complementary techniques to verify the aggregation process and study the aggregate morphology and homogeneity

To verify the presence of the aggregates, completion of the process, to check the homogeneity/polymorphism regarding the aggregated forms and study the aggregate size and morphology, some complementary techniques are recommended to use. This information can help interpreting the CD structural analysis. Thioflavin‐T (ThT) is an amyloid specific fluorescent dye that exhibits highly intensified fluorescence when bound to amyloid fibrils (Gade Malmos et al., 2017). Other types of aggregates also increase the fluorescence of ThT, however, to a lesser extent. Small aliquots (5 μL) can be withdrawn from the aggregating solution and added to typically 1 mL assay solution containing 5–10 μM ThT and the fluorescence intensity can be measured in a fluorimeter (445 nm excitation, 485 nm emission). Alternatively, ThT can be directly added to the aggregating solution at 5–20 μM concentration and the aggregation process can be followed by fluorescence measurement in a plate reader. The aggregate size, morphology, homogeneity can be studied by transmission electron microscopy (TEM) or atomic force microscopy (AFM). In the case of TEM, a simple negative staining on formvar coated copper grids and inspection at 80 or 100 kV will show the type(s) of aggregates, their size, morphology, and homogeneity (Gras et al., 2011; Winey et al., 2014). AFM can be carried out on dry samples or in solution on mica surface and gives similar data with lower resolution (Adamcik & Mezzenga, 2012; Maity & Lyubchenko, 2020).

### 2.2 Sample concentration versus pathlength

A wide range of protein concentration (0.05–50 mg/mL) can be used for CD, provided that the pathlength is properly chosen to keep the overall sample absorbance optimal in the entire wavelength range. As a rule, the product of the concentration (in mg/mL) and pathlength (in mm) should be around 0.1–0.2. Typically, 0.1 mg/mL concentration is used in a 1‐mm quartz cell (approximately 200 μL volume) and 10 mg/mL in a quartz or CaF_2_ cell of 10 μm pathlength (3–20 μL volume depending on the cell type). In a 1‐mm cell, the background absorption of the buffer (strong absorbing buffer compound or high salt concentration) might limit the usable wavelength range, while in a 100‐μm cell it is less problematic and might be no problem in a 10‐μm cell. However, short pathlength cells might have large uncertainty in the pathlength and it is important to measure their pathlength by interferometry or dilution methods (Miles, Whitmore, et al., 2005; Miles, Wien, et al., 2005).

### 2.3 Instrumental parameters, HT limitation

Normally, CD measurement of amyloid fibrils and protein aggregates require common instrumental parameters as for globular proteins or IDPs. For detailed presentation of the technique, instrumentation, measurement setup, amplitude and wavelength calibration, please, refer to the available nice reviews (Greenfield, 2006; Kelly et al., 2005). An important point is to take care of the photomultiplier voltage (HT), which should be below an instrument/detector‐specific limit. CD data with HT over the limit should be discarded and not used. The HT limit is around the 50%–60% of the maximum voltage, for example, around 600–700 V on JASCO spectropolarimeters. Typically 1 nm bandwidth is used, however, in case of high sample absorptions, 2 nm bandwidth provides higher intensity and helps to extend the usable wavelength range. Larger bandwidth is not recommended because it might distort the spectral shape. In case of continuous scanning mode, the scanning rate and integration time should be set so that the wavelength shift during the time length of the integration of a datapoint should be around the bandwidth, typically not more than 2 nm. For 50 nm/min scanning rate, a 2 s integration time is suitable. To collect data with low noise, sufficient number of scans should be accumulated. For smooth, good quality data, the overall measurement time of one spectrum should be at least 15 min (e.g., 10 scans with 50 nm/min rate in the 190–260 nm wavelength range). A data step not more than 1 nm should be used, otherwise the analysis methods will not work, we recommend a data step of 0.1 or 0.2 nm. When measuring aggregation kinetics, depending on the aggregation rate, a continuous recording of the spectra is needed, making a compromise between spectral noise and time resolution. However, spectra collected in 1–2 min will be of poor quality. To follow fast aggregation kinetics, measurement at fixed wavelength characteristic of the type of secondary structure to be followed can be used, providing sub‐minute time resolution, however, it reduces the gained structural information.

SRCD instruments, such as the one at the DISCO beamline SOLEIL Synchrotron (Giuliani et al., 2009), provide several advantages over conventional instruments for studying protein aggregates. Their light source (synchrotron radiation) produces stronger intensity in the lower wavelength range expanding the wavelength limits, and the short distance between the detector and the sample decreases the effect of light scattering. The frequent calibration makes the results reliable. Usually, cells with pathlengths below 100 μm are used, which, combined with the higher light intensity, makes possible the study of solutions containing high salt or absorbing buffers that are non‐ideal for conventional instruments. SRCD measurements require protein concentrations in the mg/mL range.

### 2.4 What baseline to be subtracted? Incomplete amyloid formation

**FIGURE 2:**
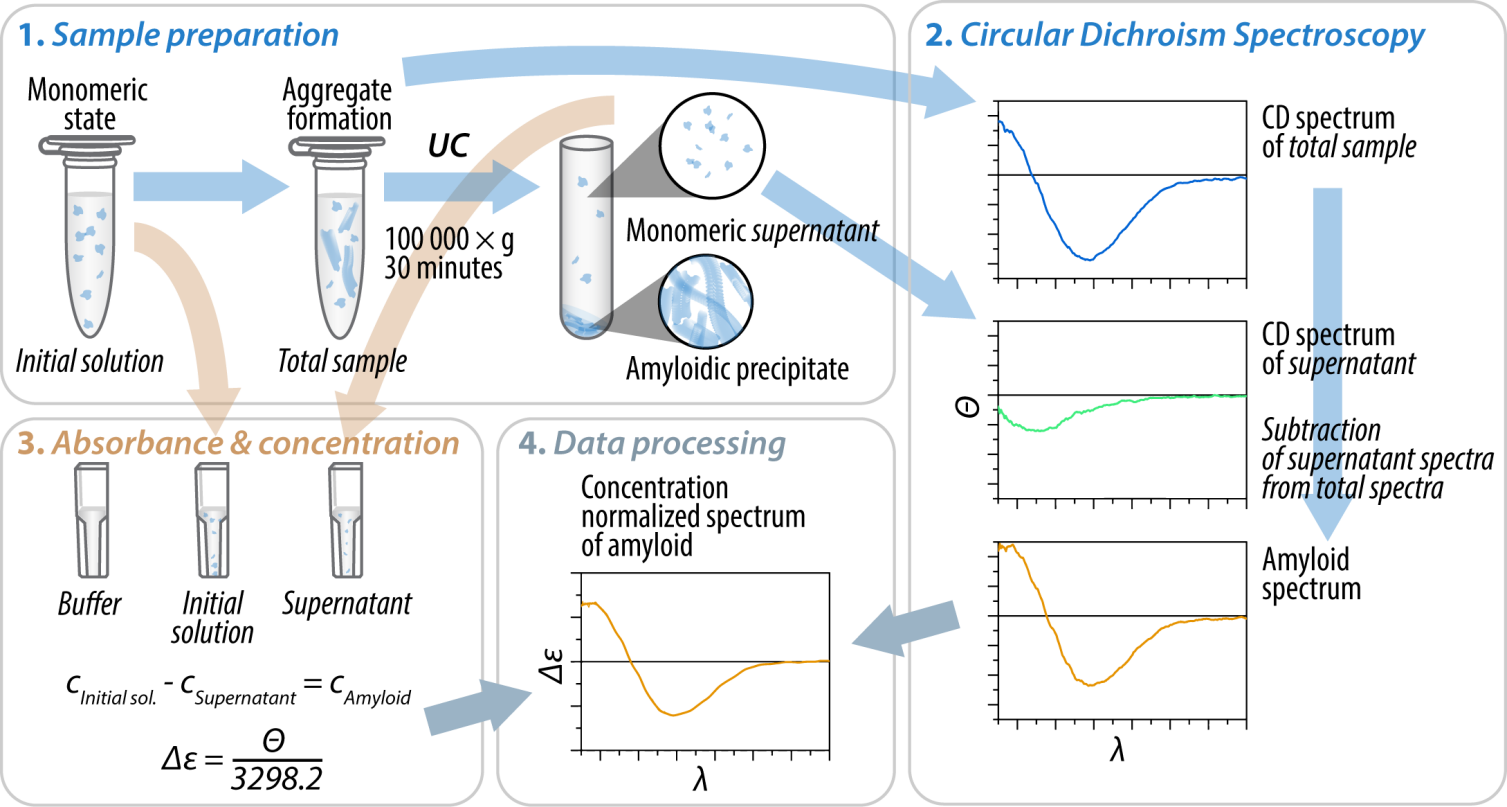
Schematic representation of the protocol for the CD and concentration measurements of aggregated samples and their proper references to obtain high‐quality, correctly normalized CD spectra.

Being an absorption spectroscopy technique, the proper background subtraction is mandatory to get the CD spectrum of the protein sample. In the case of protein aggregation, depending on the kinetics and stability of the aggregates, the sample is a mixture of monomeric molecules and aggregates. The measured CD spectrum is the average of the contribution of all the molecules in the cell. We can follow the kinetics of change of the overall average secondary structure of the protein molecules in the cell by time dependent measurements. In such a case we generally use the buffer baseline for subtraction. However, to address the structure of the aggregates (mainly in samples that reached equilibrium), we need the CD spectrum of the aggregates without the monomer contribution. By subtracting the buffer CD spectrum as baseline, we get the average CD spectrum of the total protein content of the sample. Compared to the monomers, the size of the aggregates, amyloid fibrils is much larger (except low level oligomers of small peptides), thus they can be sedimented by ultracentrifugation (~100,000 × *g* for 30 min) while keeping the monomers in solution. The CD spectrum of the resulting supernatant will be the perfect baseline to subtract all the non‐aggregate contributions from the total sample and get the CD of the aggregates/amyloid fibrils. We have to note that for normalization, we need to know the correct concentration value of the aggregate fraction (see later). Figure 2 presents the schematics of the procedure. Some researchers, after removing the supernatant from the centrifuged solution, instead of measuring the supernatant for background, re‐suspend the pellet in buffer to collect the CD spectrum of the aggregates at higher concentration, supposedly with negligible monomer contribution. However, we only recommend this procedure when the aggregate content is very low in the sample. It is difficult to properly re‐suspend the pellet, the sample will easily be inhomogeneous, contain large precipitates and might also dissociate to monomers in an uncertain amount. Such a result should rather be used only for qualitative analysis, for example, proving the presence of amyloid fibrils or aggregates in the solution. To check the possibility that small size aggregates remained in the supernatant, and if we are interested in their structural properties, the supernatant spectrum (after buffer subtraction) can be compared to the spectrum of a pure monomer solution.

### 2.5 Inhomogeneity of the aggregate solution

**FIGURE 3:**
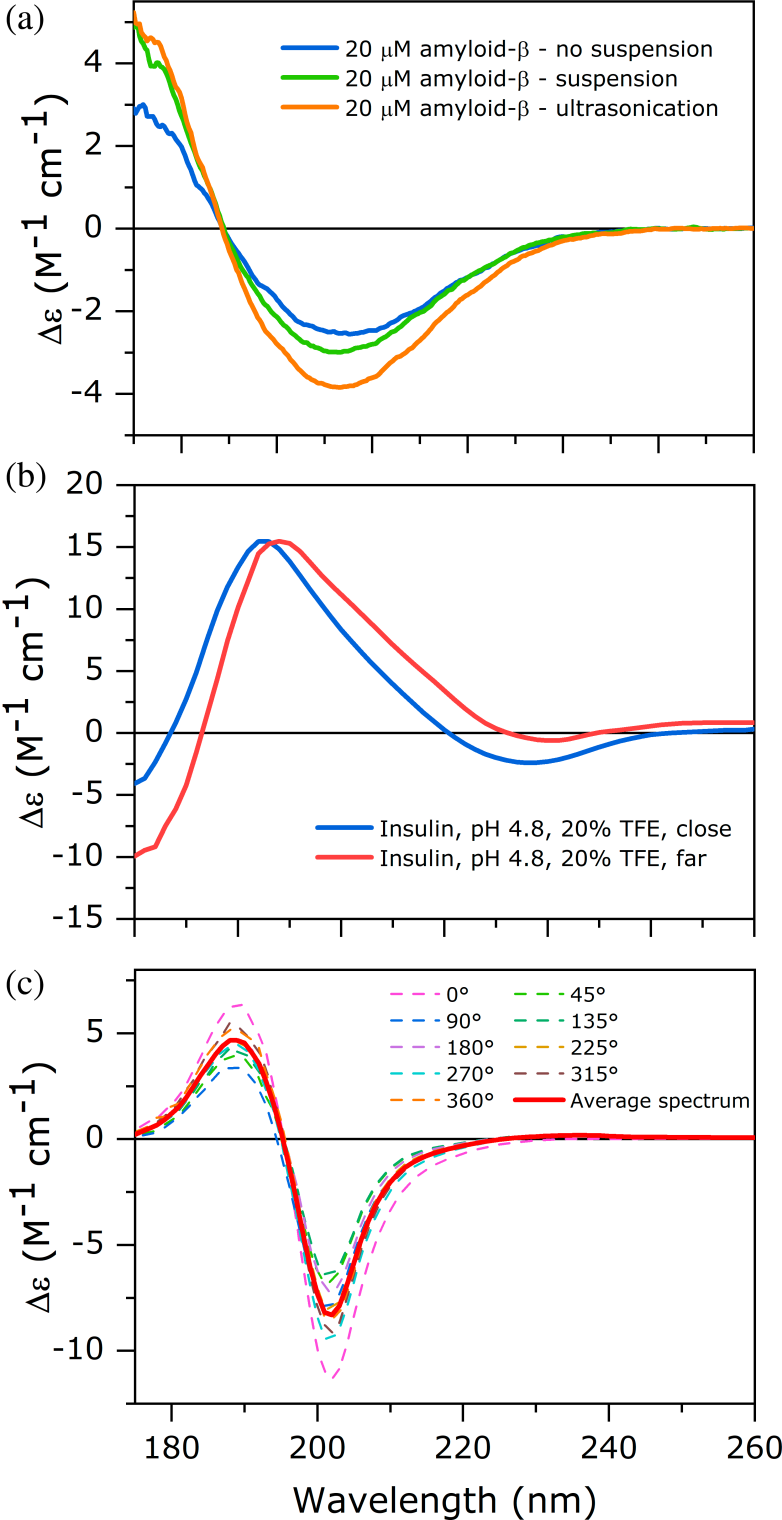
Different effects that distort the CD spectra of protein aggregates. (a) Impact of inhomogeneity on the CD spectrum. CD measurements of 20 μM amyloid‐β (1–42) amyloid fibril solution formed in a buffer of 20 mM Na‐phosphate, 100 mM NaCl, pH 7.5 after 5 days incubation at 37°C under shaking at 500 rpm. During incubation, the fibrils laterally associate and form larger aggregate particles (the solution is still transparent) making the solution inhomogeneous. When the sample was thoroughly homogenized by pipetting or slight ultrasonication, we can observe an increased CD amplitude. Ten scans were accumulated at 50 nm/min scanning rate, 2 s data integration time, 1 nm bandwidth. (b) The effect of differential light scattering on the CD spectrum. Insulin amyloid fibrils formed in the presence of 20% TFE in 20 mM Na‐acetate, pH 4.8, after incubation for 2 days at 37°C. First, CD measurement was carried out with a sample‐detector distance of 15 cm. Then the cuvette was moved close to the detector (1 cm) and the sample and baseline spectra were recorded again. In the close position, less scattered light was lost for the detector. Note the difference in the spectral shape and the upshift around 250 nm when the sample was far from the detector. (c) The effect of linear dichroism of oriented fibrillar structures on the CD spectra. Amyloidogenic GNNQQNY peptide fragment of yeast Sup35 protein shows position (angle) dependent spectra in a rotatable cell. Upon rotation, the shape and amplitude change. By collecting a series of spectra rotating the cell around, the average spectrum will provide the correct CD spectrum of the fibrils. 10 mg/mL peptide solution aggregated in water was diluted twice with water and the spectra were recorded in dismountable round‐shape CaF_2_ cell of 12.5 μM pathlength by SRCD at DISCO beamline, SOLEIL Synchrotron.

The aggregated protein samples often contain large, sometimes visible precipitates, which can be formed by further association of smaller aggregates or lateral association of amyloid fibrils in large insoluble bundles. Such precipitates are non‐transparent for the incident light and will not contribute to the CD spectrum. In the presence of them, the spectral amplitude will be shrunk, in some worse cases, the spectrum will not be different from the CD spectrum of the supernatant. In the case of an unexpectedly low spectral amplitude, we might try to homogenize the sample solution with thorough pipetting or slight ultrasonication. A thorough homogenization is recommended before every CD measurement of protein aggregates. The phenomenon is presented in Figure 3a. After CD measurement, the sample in the cuvette should be visually inspected for precipitates or sedimented material. In problematic cases, the CD spectrum can only be used for qualitative purposes, for example, to show that aggregation occurred, based on that the CD spectrum of the native protein is converted to a spectrum with an altered shape characteristic of β‐sheets or disappeared.

### 2.6 The effect of differential light scattering

Because their large size, protein aggregates might scatter the light. When the light is scattered out of the optical path, it will not reach the detector and appear as absorbance increase. Depending on the aggregate size and morphology, the scattering effect might differ for the left and right‐hand circular polarized light (called differential light scattering) and thus appear as CD signal and might significantly distort the protein CD spectrum. The intensity of scattering strongly depends on the size of the aggregates, thus a homogenization and breakage of fibrils into smaller pieces can decrease the scattering effect. Another possibility is to lose less scattered light by placing the cuvette closer to the front end of the detector. Differential scattering is often manifested by a shift of the baseline‐subtracted spectrum resulting that the flattening part of the spectrum in the 250–260 nm region will not tend to be around zero but up‐ or downshifted. The phenomenon and the effect of the change of the sample‐detector distance on the amyloid CD spectrum is presented in Figure 3b. How complicated to change the sample cell‐detector distance depends on the instrument, the geometry of the sample chamber and the type of the cell holder. The distance can be decreased to a few cm. However, changing the position of the cell might be problematic if the light beam of the instrument is not parallel and focused enough at the new position. This could be tested by control measurements with a non‐scattering protein sample and its buffer baseline at both positions. After baseline subtractions, the CD spectra of the control protein recorded in the two positions should be identical.

### 2.7 Effect of linear dichroism

Long amyloid fibrils might become oriented in the cell, especially in the case of a short pathlength as an effect of the flow upon filling the cell. Another possibility is that the peptides interact with the cell wall and deposit on the surface in an oriented manner, as was observed in the case of CaF_2_ cells with the GNNQQNY amyloidogenic peptide (Figure 3c). Usually, CD instruments are sensitive to linear dichroism (LD) in the sample. Such oriented aggregate content might affect and significantly distort the CD spectrum. The phenomenon can be detected by the change of the CD spectrum upon rotating the sample using a cuvette of cylindrical shape in an appropriate cell‐holder (rotating around the axis of the light beam). Theoretically, averaging the CD spectra collected from all angles will give the correct CD spectrum. Such samples are worth to study by linear dichroism spectroscopy, a technique not discussed here.

### 2.8 Concentration determination and normalization

To estimate the secondary structure composition, the background‐subtracted CD spectra should be normalized to the molar concentration of residues (more precisely, to the number of peptide bonds) and the pathlength. Normalized CD can be expressed either as mean residue ellipticity (MRE), or [*θ*] in units of deg. cm^2^/dmol or molar extinction coefficient difference (Δ*ε* in units of M^−1^ cm^−1^), as follows:

$${\left[\theta \right]}_{\mathrm{MRE}}=\theta /\left(10\cdot c\cdot \left(n_{r}-1\right)\cdot l\right)$$

where *θ* is the measured ellipticity in mdeg, *c* is the molar concentration of the protein, *n*_r_ is the number of residues in the molecule, and *l* is the pathlength in cm.

$$\Delta \varepsilon =\left[\theta \right]/3298.2$$

The proper normalization is crucial for accurate secondary structure estimation and spectral comparisons. Usually, the overall protein concentration of the sample is known and can be easily determined in the starting monomer protein solution. To have the concentration of the aggregate content in the sample, the protein concentration of the supernatant after ultracentrifugation (100,000 × *g*, 30 min) should be subtracted from the total concentration. Containing soluble monomers only, the concentration determination of the supernatant is not complicated either. Concentration can be determined by using the classic amino acid analysis, some sensitive color assays, or, easier, by measuring the absorption at 205, 214, or 280 nm and calculating with the respective extinction coefficients using the Beer–Lambert law:

$$c=A/\left(\varepsilon \cdot l\right)$$

where *c* is the molar concentration of the protein, *A* is the measured absorbance at the chosen wavelength and *ε* is the molar extinction coefficient (M^−1^·cm^−1^).

The molar extinction coefficient at 280 nm can be calculated from the amino acid sequence using the Protparam tool of Expasy (https://web.expasy.org/protparam). At 280 nm, the background absorbance is usually low, however, in the presence of low number or lack of Trp residues, it has limited accuracy. The advantage of measuring the absorbance at 205 and 214 nm is the higher extinction coefficients, high enough to directly measure the absorbance of the CD samples, which is often possible to calculate from the HT values during the CD measurement in conventional instruments. However, it is important to carefully measure and subtract the buffer absorbance, which can be high at these wavelengths. To correct for any possible light scattering effects, we recommended to measure the absorbance at 310 nm, where the proteins should have no contribution, and correct the 205, 214, or 280 nm absorbances with this value. The sequence‐specific extinction coefficients at 205 and 214 nm can be calculated at the BeStSel webserver (https://bestsel.elte.hu/extcoeff.php) based on the works of Anthis et al. and Kuipers et al., respectively (Anthis & Clore, 2013; Kuipers & Gruppen, 2007).

To determine accurately the concentration of a re‐suspended aggregate pellet sample, it is recommended to dissolve the aggregates in urea, GdnHCl or in some organic solvent and measure the absorbance with proper reference subtraction.

### 2.9 Secondary structure estimation

The structures of protein aggregates and amyloid fibrils are usually rich in β‐sheets. However, β‐sheet is a diverse class of protein secondary structure. β‐Sheets can differ in the parallel–antiparallel orientation of the β‐strands, the length of the composing strands and in the twisting of the sheets. Amyloid fibrils are usually characterized by a cross‐β structure where the β‐strands are oriented approximately perpendicularly to the fibril's axis. In most cases, amyloid fibrils consist of parallel β‐sheets, while antiparallel β‐sheet content of various twisting is frequently described in the structure of oligomers, prefibrillar aggregates or non‐specific aggregates. To derive the secondary structure composition of protein aggregates from their far‐UV CD spectra, suitable algorithms are needed. As it was pointed out in our previous work (Micsonai et al., 2015), most of the available algorithms fail to correctly estimate the structure of β‐structure‐rich protein samples, especially in the case of the parallel β‐sheet content of amyloid fibrils. The reason is the aforementioned structural diversity of β‐sheets, which is reflected in a large spectral diversity the algorithms cannot handle. We showed that the parallel–antiparallel orientation and the twist of the β‐sheets can be accounted for the spectral diversity and developed the ‘Beta Structure Selection’ (BeStSel) algorithm that distinguishes four different types of β‐sheet components (Table 1) and provides more accurate secondary structure estimations compared to previous tools on any structural class of proteins including β‐sheet‐rich proteins and amyloid fibrils. The algorithm is freely available at the BeStSel webserver (https://bestsel.elte.hu) (Micsonai et al., 2022), and currently is incorporated to the Spectra Manager software of JASCO. Besides BeStSel, only the CDNN (Bohm et al., 1992) and LINCOMB (Toumadje et al., 1992) algorithms distinguish parallel and antiparallel β‐sheets. A collection of numerous algorithms are available at the DICHROWEB server (Whitmore & Wallace, 2004), however, they all suffer from the problem of predicting β‐sheet content correctly for high parallel β‐sheet content and twisted β‐sheets (Micsonai et al., 2015).

**TABLE 1:** Structural components of BeStSel.^a^

Structural component	Description of the component	Related DSSP component
Helix1	Regular α‐helix (middle part of α‐helices)	H
Helix2	Distorted α‐helix (2‐2 residues at ends of α‐helices)	H
Anti1	Antiparallel β‐sheet (left‐hand twisted)	E
Anti2	Antiparallel β‐sheet (relaxed, slightly right‐hand twisted)	E
Anti3	Antiparallel β‐sheet (right‐hand twisted)	E
Parallel	Parallel β‐sheet	E
Turn	Turn (as defined by DSSP)	T
Other	3_10_‐helix, π‐helix, β‐bridge, bend, loop/irregular and invisible regions of the structure	G, I, S, B, O

^a^ The BeStSel webserver is capable of a reliable structure estimation from CD data. Secondary structure elements are derived from DSSP categories as shown in the third column. DSSP is the standard algorithm to assign secondary structure to the amino acids of a protein, based on the atomic‐resolution coordinates (Kabsch & Sander, 1983). In BeStSel, α‐helix elements (H) are divided into two categories. Regarding β‐sheets (E), antiparallel β‐sheets are divided into three categories by their twist and the fourth β‐sheet element is the parallel β‐sheet (Micsonai et al., 2015, 2021). The ‘Other’ component mainly contains the disordered and irregular structures and some rare secondary structure elements are also assigned here.

Considering the above statements, BeStSel is currently the most suitable method for the analysis of protein aggregates including amyloid fibrils. For a critical assessment of structure estimation, a thorough quality check should be carried out on the CD spectra to be analyzed and the secondary structure result should also be inspected.

#### 2.9.1 Quality control, check for amplitude shrinking or abnormal spectral amplitude

**FIGURE 4:**
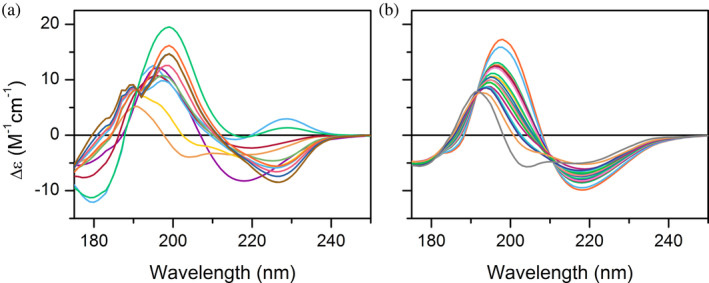
Calculated CD spectra of amyloid fibrils having available 3D‐structure in the PDB. The spectra of (a) amyloid fibrils containing antiparallel β‐sheet and (b) fibrils with parallel β‐sheet structures were calculated by the appropriate linear combination of the basis spectra of the BeStSel algorithm. Amyloid spectra differing significantly in shape or amplitudes from these CD spectra should be double checked for correct normalization, presence of disturbing effects and abnormal contributions and their secondary structure estimation result might be considered unreliable.

The normalized CD spectrum should be inspected for its amplitude to exclude the possibility of shrinking/signal loss. It might be difficult, because the shape, amplitude, and the area of the spectra depend on the secondary structure composition and on the wavelength range used. α‐Helical proteins give the highest amplitude and area, while the spectral components of relaxed antiparallel and disordered structures exhibit lower amplitudes and area. We propose a checkup for the area under the CD curve that is normalized to Δ*ε* values, as the sum of the absolute values of the CD signal at integer nm wavelengths in the measured range. To determine the reasonable spectral areas, first we calculated these values for all the proteins of the SP175+ CD reference dataset used in our previous work (Micsonai et al., 2015). However, this dataset mainly contains globular proteins, only a part of them has high β‐sheet content and only two of them are amyloid fibrils. At present, there are over 100 amyloid 3D‐structures deposited in the PDB, however, most of them have no CD spectra available. We have reconstructed the CD spectra of a large selection of them by the linear combination of the basis spectra of the BeStSel method according to the secondary structure compositions derived from the PDB structures (Figure 4). The CD spectra were calculated for all the amyloid structures containing antiparallel β‐sheets and for selected spectra containing parallel β‐sheet (this is the most common amyloid structure). The calculated area ranges are shown in Table S1. We suggest to check the area of your spectrum before secondary structure analysis by BeStSel. If the area of the spectrum normalized in Δ*ε* is lower than the expected limit, there is a high chance that the spectral amplitude is decreased or the spectrum is not normalized properly. In such case, the secondary structure estimation is not reliable. Only qualitative characterization of the spectrum should be given. To avoid shrinking of CD the spectrum, see Section 2.5.

The normalized CD spectrum should also be checked whether its amplitude is not higher than 20 M^−1^ cm^−1^, or the area is not over the range shown in Table S1. In case of extremely high amplitudes, there might be a problem with the concentration determination and normalization or there might be an unexpected high amplitude component present. An abnormally high amplitude and strange spectral shape can be the result of differential light scattering, linear dichroism, or might originate from for example, the stacking of aromatic side‐chains or, in the case of short peptides, from a specific amino acid composition that is very different from the typical proteins' (e.g., high percentage of aromatic residues or proline, presence of disulfides). In the case of large, mainly three‐dimensional aggregates with sizes reaching the wavelength of the exciting light, a psi‐type CD spectrum might be observed which is the result of long‐range interactions among chromophores and does not reflect the secondary structures of the polypeptide chain (Kim et al., 1986). Abnormally large amplitudes were observed for some aggregates of insulin (Muta et al., 2014). In the case of strong differential light scattering, or aromatic stacking, usually the near‐UV CD spectrum in the 250–320 nm wavelength range also has a high amplitude and can serve as further indication. Such effect was observed on the fibrillary aggregates of Trp‐cage miniprotein (Kardos et al., 2015). BeStSel cannot handle correctly the abnormal spectral shape and amplitude (none of the algorithms do). With abnormal components, the spectrum can be used as “fingerprint” for the corresponding amyloid fibrils/aggregates but the secondary structure analysis will not be correct. To avoid light scattering, and the effect of linear dichroism see Sections 2.6 and 2.7.

#### 2.9.2 QC for light scattering, correction

After proper baseline subtraction, the amplitude of the CD spectrum should converge to zero around 260 nm. If there is an up‐ or downshift, it usually indicates the possible presence of a light scattering effect, or might be caused by nucleic acid contamination (see Section 2.9.3). To avoid light scattering, see Section 2.6. As correction for minor shifts, the entire spectrum can be shifted up or down with a constant to reach zero at 260 nm. The corrected spectrum can be used for secondary structure analysis.

#### 2.9.3 QC, nucleic acid contaminations, CD, and absorbance profile check

Another reason for the non‐zero CD amplitude at 260 nm might be a nucleic acid contamination, which might occur if the protein has affinity for RNA or DNA, or as a result of insufficient isolation/purification. The best way to verify this is to check the absorbance spectrum in the 200–320 nm wavelength range. Nucleic acids have a characteristic absorbance around 260 nm, where the protein absorption is relatively low. Another sign of contamination is when the concentration measurements by photometry at 205, 214, and 280 nm differ, usually the 280 absorbance gives much higher concentration than expected. Unfortunately, nucleic acids exhibit strong CD bands in the wide wavelength range of 175–320 nm and their contamination distort the entire far‐UV CD spectrum. The solution is to get rid of the contamination by further purification, for example, using a column for specific nucleic acid binding. It might be difficult in case of strong nucleic acid binding affinity of the protein, but it is essential.

#### 2.9.4 Secondary structure analysis of CD spectra of protein aggregates by the BeStSel method

Normalized CD spectra or measured, baseline‐corrected CD spectra with concentration and residue number data can be uploaded to the BeStSel webserver at https://bestsel.elte.hu. A detailed tutorial is downloadable on all the functions of the site and explained in detail in previous publications (Micsonai et al., 2015, 2018, 2021, 2022). Data can be uploaded as text file or can be copied into the window in two data columns. Column separator can be space, tab, comma, or semicolon. In the case of text files, the software will ignore any header and upload the data. CD spectra saved in text format on various CD instruments will be suitable for the system. Data at wavelength steps of 0.1, 0.2, 0.5, or 1 nm are accepted in increasing or decreasing order. Data units have to be chosen in a box. Note that the numeric format uses dot as decimal point. The upload is protected by a captcha against malicious use. After upload, a ‘Data Examination’ page appears where we can check the proper upload of the data. Data will be shown in normalized form converted to Δ*ε*. After checking the data, secondary structure is calculated with a single click and shown in a savable graphical image with the estimated secondary structure percentages, experimental and fitted spectra including the residuals between them, RMSD and NRMSD values. Below the results, the output format can be changed for the user's convenience. For data processing by the users, all data can be shown in text format to copy out, or saved as .csv file.

In BeStSel, eight secondary structure components are distinguished, including four types of β‐sheets (left‐hand twisted, relaxed and strongly right‐hand twisted antiparallel β‐sheets named as Anti1, Anti2, Anti3, respectively, and parallel β‐sheet), each having distinct spectral contribution (Table 1). Compared to the 3–6 components estimated by other algorithms, this increased information provides opportunity for structural comparison of protein aggregates and amyloid fibrils. Multiple spectra can also be analyzed with a few clicks, which is especially useful when measuring aggregation kinetics or conformational changes as a function of temperature or in the presence of additives.

Regarding RMSD and NRMSD (normalized RMSD) of spectral fitting, a large deviation in the fitting might indicate a large expectable error in the structure estimation. However, there is no strong correlation with the accuracy of the secondary structure estimation in the case of BeStSel or any other algorithms (Micsonai et al., 2015). Moreover, depending on the algorithm, the RMSD (and also NRMSD) values differ significantly, even analyzing the same CD spectrum. A perfect fit does not mean a perfect secondary structure estimation. Extreme examples are the CDSSTR method (Sreerama & Woody, 2000) always providing a perfect fit, even when it is far from the right estimation, and K2D2 (Perez‐Iratxeta & Andrade‐Navarro, 2008), which can give a reasonable estimation even when the RMSD of the spectral fit is large. However, even in the lack of direct relationship with the structure estimation error, it is important to check the deviations between the experimental and fitted spectra and interpret them properly. For BeStSel, we calculated the RMSD and NRMSD values on 93 proteins (Figure S1), 73 proteins of the SP175+ dataset and 20 more proteins of the PCDDB database (Micsonai et al., 2015), all having high quality, smooth CD spectra. Figure S1 shows that the RMSD values depend on the wavelength range while NRMSD values are more uniform, however, both show a wide distribution on the various proteins. Our recommendation is if the RMSD and NRMSD of the fitting of the analyzed spectra are over the limits presented by the 93 proteins, the results should be handled with caution. A warning limit for NRMSD is approximately 0.06 for any wavelength range, while for RMSD, it is 0.6 in the 175–250 nm wavelength range and decreasing to 0.2 by narrowing the wavelength range to 200–250 nm. We have to note that the RMSD highly depends on the spectral noise. If the spectrum is noisy (e.g., insufficient number of scans were accumulated), the fluctuations in the spectrum will add up as a large RMSD, even if the structure estimation is correct, because the fitted spectra will always be fairly smooth.

The secondary structure results finally should be checked whether they are realistic. Usually, even in the highly ordered amyloid fibrils, the amyloid core does not span the entire sequence, there are turn regions and disordered, flanking parts. A result containing only a single secondary structure element in 100% or 1–2 structural components with no *Turn* and *Other* component, should be warning. Such result is often caused by an abnormal amplitude (check normalization, concentration, presence of distorting effects, etc.) or unexpected spectral contribution.

### 2.10 Case studies on structural analysis of protein aggregates

Here, in some case studies, we present the applicability and effectiveness of CD spectroscopy for structural analysis of protein aggregates. These examples enlighten the large spectral diversity observable even for a single protein, which is a reflection of the structural variety of the protein aggregates that can be formed under different conditions. In all cases, CD spectra are reproducible and specific for the protein and the experimental conditions.

**FIGURE 5:**
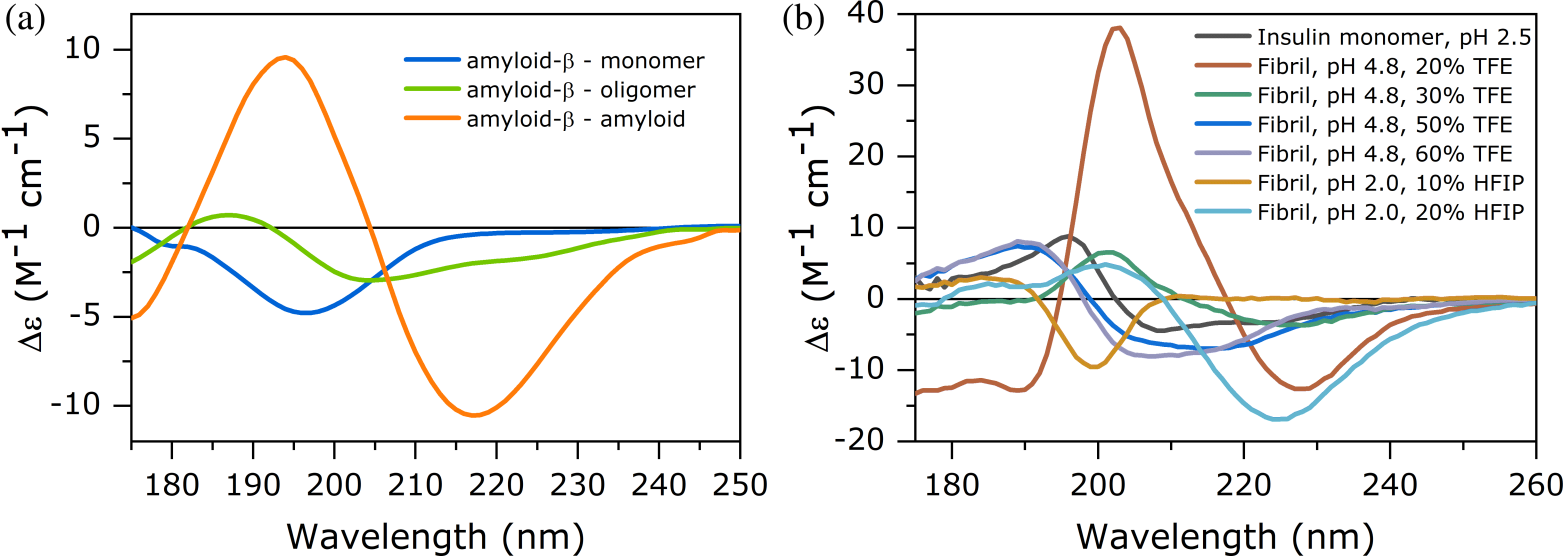
Secondary structure analysis of Aβ (1–42) and insulin aggregates. (a) CD spectra of amyloid‐β (1–42) peptide in different conformational states. The freshly solubilized monomer peptide at pH 7.5 exhibits a spectral shape characteristic of disordered proteins. Incubating the sample at 4°C for 5 days, the solution will contain Aβ oligomers and prefibrillar aggregates with low amount of remaining monomers. At pH 2.0, 37°C, homogeneous amyloid fibril solution, free of other type of aggregates, can be formed with parallel β‐sheet secondary structure. These measurements were carried out by SRCD at DISCO beamline, SOLEIL Synchrotron. Secondary structure estimation was carried out with BeStSel and presented in Table S2. (b) Insulin aggregates characterized by diverse CD spectral shape formed under different conditions. 5 mg/mL insulin samples were incubated for 5 days under the indicated conditions with 500 rpm agitation at 37°C. SRCD spectra were recorded in a 12 μm CaF_2_ cell at the DISCO beamline, SOLEIL Synchrotron. Samples were gently ultrasonicated before filling into the cell. We have to note, that the sample prepared at pH 4.8 in the presence of 20% TFE showed abnormal, extremely high amplitude for a yet unknown reason. Its structural analysis should be treated with caution. Secondary structure analysis by BeStSel is presented in Table S3.

Alzheimer's amyloid‐β (1–42) peptide is highly prone to aggregation and can form different types of aggregates from various sizes of oligomers to prefibrillar aggregates, and amyloid fibrils with different morphology. A homogeneous amyloid fibrils solution with parallel β‐sheet structure can be formed at pH 2. Figure 5a shows the CD spectrum of these fibrils along with the spectra of a monomeric solution and a sample aggregated at low temperature and physiological pH, which is favorable for oligomer formation (Stine Jr et al., 2003). The Aβ amyloid fibril sample is perfect to compare the capability of the available algorithms for secondary structure estimation to analyze aggregate and amyloid fibril CD spectra because the 3D‐structure of this amyloid form has been solved by solid‐state NMR and deposited in the PDB. The structure contains 47.6% parallel β‐sheet. Table S2 presents the results of the secondary structure analysis by the most frequently used algorithms in comparison to the PDB structure. The results revealed that SELCON, CDSSTR, LINCOMB, K2D3 and CAPITO erroneously estimate high, 47%–65% α‐helical and low β‐sheet content. CONTIN performed somewhat better, predicted 23% helix and 32% β‐sheet. Besides BeStSel, LINCOMB, and CDNN algorithms distinguish antiparallel and parallel β‐sheets, all identified the presence of the parallel component in the Aβ fibrils. However, only the results of CDNN and BeStSel showed acceptable accuracy (Table S2). We recommend the use of these two algorithms for analysis of protein aggregates and do not recommend any other one. BeStSel provides higher information content by its four β‐sheet components, thus it has more capability to make distinctions between variants of fibrils and in identifying fibril types.

Native human insulin under physiological conditions exhibits α‐helical structure. Its aggregation can be easily induced by high temperature, extreme pH, or the presence of alcohols. Depending on the experimental conditions, it can form aggregates and amyloid fibrils of various secondary structure composition and morphology (Muta et al., 2014). Figure 5b and Table S3 show the CD spectra and corresponding secondary structures estimated by the BeStSel method revealing the enrichment of β‐structures in all the fibrillary forms, however, depending on the conditions, parallel or various antiparallel β‐sheet‐rich structures can also be formed. At pH 4.8 in the presence of 20% TFE, a characteristic, abnormally high positive amplitude is observed, which is outside of the expected amplitudes for BeStSel and the estimated secondary structure values are non‐valid. Qualitatively, the spectral shape is similar to left twisted or relaxed antiparallel β‐sheets and FTIR examination also suggested the presence of antiparallel‐β structure (Muta et al., 2014).

### 2.11 Recent advances achieved by CD spectroscopy in the field of protein aggregation

To show the versatility of CD spectroscopy on the study of protein aggregation and provide ideas for potential future users we review some of the recent advancements in the protein aggregation field achieved with the help of CD spectroscopy.

Buchner's group investigated the aggregation mechanism in antibody light (AL) chain amyloidosis. CD spectroscopy together with BeStSel analysis showed a structural rearrangement while keeping the highly ordered structure in oligomers before amyloid formation. The observed multi‐step structural transitions offer opportunities for therapeutic intervention (Kazman et al., 2021). Zheng and co‐workers observed that the short amyloidogenic fragment from yeast prion protein Sup35 is capable of cross‐seeding the amyloid formation of Aβ peptide and hIAPP indicating the possibility of pathological links between misfolding diseases and the role of amyloid polymorphism. The authors followed the amyloid formation and secondary structure changes by CD spectroscopy and BeStSel analysis (Zhang et al., 2021). Lee and co‐workers studied the diverse structural conversion and aggregation pathways of Alzheimer's amyloid‐β (1–40). The sensitivity of secondary structure determination by CD and BeStSel let them describe intermediate states and transitions (Lin et al., 2019). SRCD was used as complementary technique to MD and cryo‐EM to investigate the hierarchical heterozipper β‐sheet of amyloid peptides by Dong and co‐workers (Song et al., 2022). Rosetti et al. (2024) studied the inhibitory effect of Pro‐Phe‐Phe tripeptide stereoisomers on insulin amyloid formation and showed by CD and ThT assays that the homochiral peptide is the effective inhibitor of fibrillization.

Freeze–thaw and lyophilization induced alterations in mAb therapeutics was studied on trastuzumab and five commercialized biosimilars by CD spectroscopy and secondary structure analysis (Dash & Rathore, 2021). Brito and co‐workers used CD and BeStSel to demonstrate the formation of β‐structure upon working out a protocol for transthyretin aggregation to study amyloid fibril disruption (Ferreira et al., 2021). Konig et al. (2023) studied the nanostructure and stability relationship of self‐assembled peptide fibers by CD spectroscopy, small‐angle neutron/X‐ray scattering and MD simulations.

## 3 CONCLUSIONS

**FIGURE 6:**
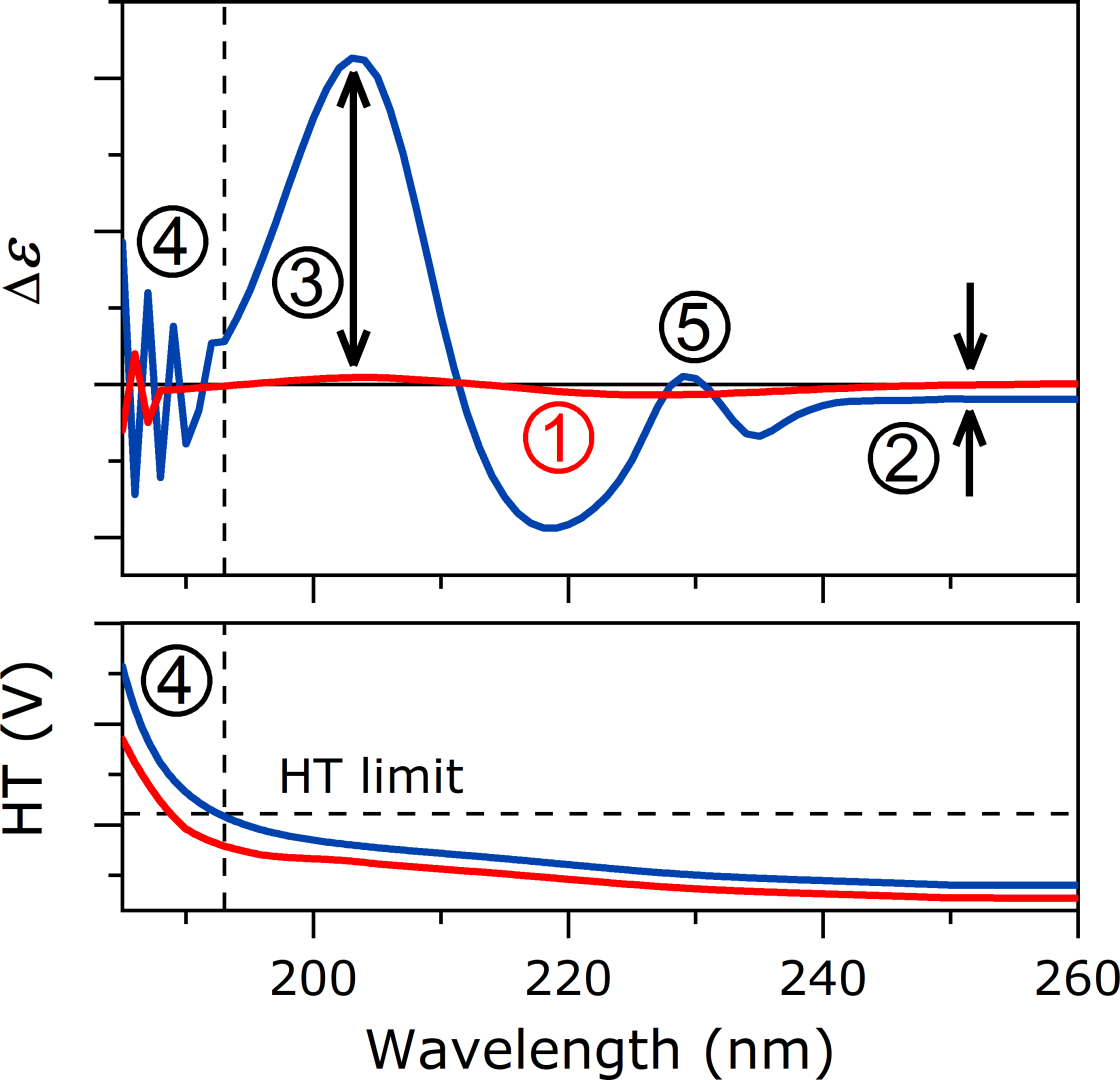
Quick check‐up of the CD spectra of protein aggregates. Numbers 1–5 point out the critical issues, low amplitude, spectral shift, extreme amplitude, noisy spectrum above (or below) the HT cutoff and presence of extra contributions, respectively, which might prevent a reliable structural analysis. Table 2 presents the quick troubleshooting for these problems. These aberrant spectral features might be informative for researchers willing to study the native state of general proteins to recognize unwanted protein aggregation or incorrect experimental/instrumental setup.

CD spectroscopy is a widely used standard method in protein science. The aim of our work is to provide an exhaustive protocol for secondary structure analysis by CD of protein aggregates and amyloid fibrils. The unique characteristics of these species make their study particularly challenging. We investigated several amyloidogenic proteins under various conditions to identify potential sources of error and suggest possible solutions. Manifestations of these key factors in the spectra are summarized in Figure 6 and Table 2 provides a quick troubleshooting guide for recording CD spectra.

**TABLE 2:** Quick troubleshooting for CD spectra.

	Problem	Solution
1	Spectra with low amplitude	Check concentration and normalization Homogenize the sample with thorough pipetting or slight ultrasonication
2	Up‐ or downshift of the spectrum	Check baseline subtraction Homogenize the sample, ultrasonication might help to decrease the size of aggregates Place the sample close to the detector Purify the sample from nucleic acid or other contaminations
3	Extreme amplitude	Check concentration and normalization Homogenize the sample with thorough pipetting or slight ultrasonication Place the sample close to the detector Might be caused by LD effect. Rotate the sample and average data Might be a valid spectral component, secondary structure estimation results are questionable
4	Noisy spectrum/HT limit	Discard data where HT is over the limit. To reach lower wavelengths, decrease salt concentration and/or pathlength with proper protein concentration Noise within usable HT range: reduce the buffer concentration and/or use higher protein concentration and/or use shorter pathlength cuvette. Set instrumental parameters properly: lower scanning rate, longer response time, open the slit width to 2 nm; accumulate more scans
5	Unknown contribution	In case of contamination, purify the sample, however, it may originate from an intrinsic contribution such as that of aromatic residues

The protocol provides detailed instructions for sample preparation and outlines the critical conditions to consider, such as pH, ionic strength, temperature, and additives. Additionally, we describe the recommended instrument parameter settings to ensure the collection of data suitable for quantitative secondary structure estimation. The protocol addresses potential measurement issues, such as sample inhomogeneity, differential light scattering and linear dichroism and offer troubleshooting strategies to mitigate these effects.

We emphasize the importance of data normalization and provide precise guidelines for executing this process correctly. Several quality control aspects are discussed to ensure that generated/recorded data are appropriate for structure estimation and publication. We also introduce software tools to estimate secondary structure content from CD spectra with particular focus on the BeStSel method, which is especially effective for analyzing protein samples rich in β‐structures.

We also mention complementary techniques that can be used to investigate the kinetics of aggregate formation, the size, morphology, and polymorphism of aggregates thus providing a complete characterization to help the interpretation of the results of the structural studies.

High‐quality case studies are presented to demonstrate the effectiveness of CD spectroscopy to study the structure of protein aggregates. Our work aims to facilitate the correct application of CD spectroscopy, thereby promoting its use in both basic and applied research, including drug development.

## AUTHOR CONTRIBUTIONS

**József Kardos:** Conceptualization; investigation; writing – original draft; methodology; validation; visualization; writing – review and editing; supervision; resources; funding acquisition. **Márton Péter Nyiri:** Investigation; writing – original draft; writing – review and editing; visualization. **Éva Moussong:** Investigation; writing – original draft; writing – review and editing; visualization. **Frank Wien:** Conceptualization; investigation; writing – review and editing; methodology. **Tamás Molnár:** Investigation; writing – review and editing. **Nikoletta Murvai:** Investigation; writing – original draft; writing – review and editing. **Vilmos Tóth:** Visualization; writing – review and editing; investigation. **Henrietta Vadászi:** Investigation; writing – review and editing. **Judit Kun:** Investigation; writing – review and editing. **Frédéric Jamme:** Conceptualization; methodology; writing – review and editing. **András Micsonai:** Conceptualization; investigation; funding acquisition; writing – original draft; writing – review and editing; visualization; methodology; supervision; software; validation.

## CONFLICT OF INTEREST STATEMENT

The authors declare no conflicts of interest.

## Supporting information


**TABLE S1.** Expected area of the CD spectra.
**TABLE S2:** Secondary structure analysis of the CD spectra of amyloid, oligomer and monomer form of Aβ (42) peptide using various algorithms.
**TABLE S3:** Structural analysis of the different forms of insulin by SRCD.
**FIGURE S1:** Spectral fitting deviations of the BeStSel method.

## Data Availability

The data that supports the findings are available in the Protein Circular Dichroisn Database (PCDDB) or upon request, provided by the authors.
