# Supplementary material for: Guide to the structural characterization of protein aggregates and amyloid fibrils by CD spectroscopy
Source: Protein Sci. 2025 Feb 19;34(3):e70066. doi: 10.1002/pro.70066 (PMC11836901; doi:10.1002/pro.70066)
Supplement: Supplementary file 1 — TABLE S1. Expected area of the CD spectra. TABLE S2: Secondary structure analysis of the CD spectra of amyloid, oligomer and monomer form of Aβ (42) peptide using various algorithms. TABLE S3: Structural analysis of the different forms of insulin by SRCD. FIGURE S1: Spectral fitting deviations of the BeStSel method. [file PRO-34-e70066-s001.pdf]

Supplementary Material

Guide to the structural characterization of protein aggregates and amyloid fibrils by CD spectroscopy

József Kardos, Márton Péter Nyiri, Éva Moussong, Frank Wien, Tamás Molnár, Nikoletta Murvai, Vilmos Tóth, Henrietta Vadászi, Judit Kun, Frederic Jamme, András Micsonai

Table S1. Expected area of CD spectra[a]

WL-range (nm)	Min. area	Max. area
175-250	77	526
180-250	64	506
185-250	54	470
190-250	41	398
195-250	24	353
200-250	20	270

[a]Minimum and maximum spectra areas were calculated on the SP175 CD reference dataset, additional amyloid fibril spectra and on the calculated CD spectra for amyloid fibril structures deposited in PDB (Figure 6). Areas were calculated as the sum of the absolute values of $\Delta\varepsilon$ at integer wavelengths in the indicated wavelength range. CD spectra with areas beyond these limits should be inspected carefully for normalization problems, shrinking, artefacts or abnormal behavior and their quantitative secondary structure estimation might not be acceptable.

Table S2. Secondary structure analysis of the CD spectra of amyloid, oligomer and monomer form of Aβ(42) peptide using various algorithms[a]

		Abeta 1-42 amyloid											
Algorithm	WL Range	H	B	TO	H1	H2	A1	A2	A3	A	P	T	O
PDB[b]		0.000	0.476	0.524	0.000	0.000	0.000	0.000	0.000	0.000	0.476	0.000	0.524
BeStSel	175-250	0.074	0.437	0.489	0.051	0.023	0.000	0.000	0.000	0.000	0.437	0.000	0.489
BeStSel	190-250	0.072	0.450	0.479	0.047	0.025	0.000	0.000	0.000	0.000	0.450	0.000	0.479
BeStSel	200-250	0.082	0.526	0.392	0.068	0.014	0.000	0.000	0.024	0.024	0.502	0.000	0.392
		H	B	TO	H1	H2	S1	S2	T	O			
PDB		0.000	0476	0.524	0.000	0.000	0.381	0.095	0.000	0.286			
SELCON3[c]	190-240	0.558	0.262	0.101	0.357	0.201	0.061	0.041	0.111	0.241			
SELCON3[d]	190-240	0.525	0.206	0.066	0.348	0.177	0.029	0.037	0.193	0.261			
CONTIN	190-240	0.233	0.320	0.390	0.233	0.000	0.320	0.070	0.147	0.229			
CDSSTR	190-240	0.475	0.297	0.249	0.291	0.184	0.113	0.136	0.124	0.156			
		H	B	TO	H	A	P	T	O				
PDB		0.000	0.476	0.524	0.000	0.000	0.476	0.000	0.524				
LINCOMB	190-240	0.648	0.000	0.352	0.648	0.000	0.000	0.000	0.352				
CDNN	180-260	0.094	0.409	0.540	0.094	0.021	0.388	0.030	0.510				
		H	B	TO									
PDB		0.000	0.476	0.524									
K2D3	190-240	0.592	0.048	0.360									
CAPITO	195-250	0.990	0.320	0.310									

		Abeta 1-42 oligomer											
		H	B	TO	H1	H2	A1	A2	A3	A	P	T	O
BeStSel	175-250	0.147	0.275	0.578	0.067	0.080	0.000	0.048	0.170	0.218	0.057	0.130	0.448
BeStSel	190-250	0.162	0.274	0.564	0.082	0.080	0.000	0.048	0.133	0.181	0.093	0.142	0.422
BeStSel	200-250	0.153	0.288	0.559	0.082	0.071	0.000	0.000	0.184	0.184	0.104	0.144	0.415
		H	B	TO	H1	H2	S1	S2	T	O			
SELCON3[c]	190-240	0.149	0.306	0.543	0.050	0.098	0.193	0.113	0.168	0.376			
SELCON3[d]	190-240	0.149	0.253	0.595	0.066	0.083	0.153	0.1	0.228	0.367			
CONTIN	190-240	0.176	0.240	0.584	0.078	0.098	0.141	0.099	0.231	0.353			
CDSSTR	190-240	0.076	0.291	0.627	0.011	0.065	0.169	0.122	0.285	0.342			
		H	B	TO	H	A	P	T	O				
LINCOMB	190-240	0.190	0.000	0.811	0.190	0.000	0.000	0.358	0.453				
CDNN	180-260	0.210	0.470	0.526	0.210	0.366	0.104	0.216	0.310				
		H	B	TO									
K2D3	190-240	0.047	0.144	0.809									
CAPITO	195-250	0.592	0.048	0.360									

		Abeta 1-42 monomer											
		H	B	TO	H1	H2	A1	A2	A3	A	P	T	O
BeStSel	175-250	0.000	0.313	0.687	0.000	0.000	0.000	0.119	0.194	0.313	0.000	0.217	0.470
BeStSel	190-250	0.000	0.326	0.674	0.000	0.000	0.000	0.112	0.214	0.326	0.000	0.181	0.493
BeStSel	200-250	0.000	0.269	0.731	0.000	0.000	0.000	0.000	0.269	0.269	0.000	0.223	0.508
		H	B	TO	H1	H2	S1	S2	T	O			
SELCON3[c]	190-240	0.110	0.241	0.643	-0.012	0.122	0.103	0.138	0.204	0.439			
SELCON3[d]	190-240	0.133	0.231	0.631	0.040	0.093	0.118	0.113	0.209	0.422			
CONTIN	185-240	0.067	0.274	0.660	0.001	0.066	0.158	0.116	0.232	0.428			
CDSSTR	190-240	0.010	0.360	0.615	-0.009	0.019	0.229	0.131	0.243	0.372			
		H	B	TO	H	A	P	T	O				
LINCOMB	190-240	0.035	0.000	0.965	0.035	0.000	0.000	0.454	0.510				
CDNN	180-260	0.068	0.124	0.796	0.068	0.098	0.026	0.275	0.521				
		H	B	TO									
K2D3	190-240	0.030	0.161	0.809									
CAPITO	195-250	0.040	0.300	0.700									

[a]The table shows the estimated secondary structure contents on the basis of their own, distinct components of the algorithms and the summed-up values for Antiparallel and Parallel, Helix, β-sheet, Turn+Others for comparison. [b]Calculated via BeStSel webserver using PDB entry 2BEG. [c]secondary structure content provided by SELMAT3 [1]. the Matlab version of SELCON3 using SP175 reference dataset [2]. [d]secondary structure content provided by original version of SELCON3, as well as CONTIN, and CDSSTR by CDPro package and SMP56 reference dataset [3] for 6 structural components. LINCOMB [4] and CDNN [5] distinguishes antiparallel and parallel β-sheets, while K2D3 [6] and Capito [7] only accounts for 3 components (helix, β-sheet and others). BeStSel [8,9] distinguishes 8 secondary structure elements as show in Table 1. Only CDNN and BeStSel provides acceptable estimation on the amyloid fibrils, however, CDNN has limited availability for the community.

Table S3. Structural analysis of the different forms of insulin by SRCD

Sample	Helix1	Helix2	Anti1	Anti2	Anti3	Parallel	Turn	Others
Insulin monomer, pH 2.5	26.6%	24.1%	0%	0%	0%	0%	13.3%	35.9%
Fibril, pH 4.8, 20% TFE	~~0%~~	~~21.6%~~	~~55%~~	~~23.3%~~	~~0%~~	~~0%~~	~~0%~~	~~0%~~
Fibril, pH 4.8, 30% TFE	0%	5.5%	2.3%	10.7%	0%	31.1%	18%	32.4%
Fibril, pH 4.8, 50% TFE	22.2%	11.5%	0%	0%	15.6%	9.5%	6.8%	34.4%
Fibril, pH 4.8, 60% TFE	25.4%	8.3%	0%	0%	19.9%	1.3%	1.9%	48.3%
Fibril, pH 2.0, 10% HFIP	0%	0%	0%	6.5%	22.1%	0%	25.5%	45.9%
Fibril, pH 2.0, 20% HFIP	24%	0%	4.1%	0%	0%	50.8%	17.8%	3.3%

[a]Secondary structures were estimated by the BeStSel method in the 175-250 nm wavelength range [9] and are consistent with previous results produced by CD and FTIR spectroscopy [10]. We have to note, that the sample formed at pH 4.8 in the presence of 20% TFE showed abnormal, extremely high amplitude, the estimated value are not reliable, the estimated 0% *Turn* and *Other* component is a warning sign.

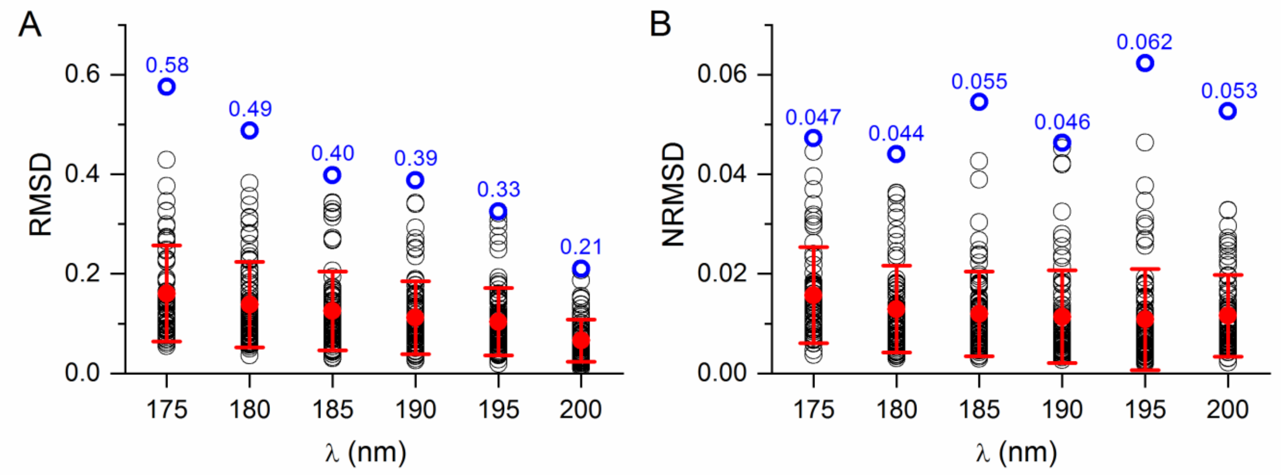

Supplementary Figure S1. Spectral fitting deviations of the BeStSel method. RMSD (A) and NRMSD (B) values of fittings to the high quality CD spectra of 93 proteins deposited in the PCDDB database [11], which have known high-resolution structures deposited in the PDB. 73 of the spectra are from the SP175+ reference set and 20 are from a set of "challenging" proteins [9]. Fittings were executed in the wavelength ranges of λ(nm)-250 nm. The averages ± SD are shown in red and the maximal deviations with their numerical values are shown in blue.

References for supporting material

1. Lees JG, Miles AJ, Janes RW, Wallace BA. Novel methods for secondary structure determination using low wavelength (VUV) circular dichroism spectroscopic data. BMC Bioinformatics 2006;7:507.

2. Lees JG, Miles AJ, Wien F, Wallace BA. A reference database for circular dichroism spectroscopy covering fold and secondary structure space. Bioinformatics 2006;22(16):1955-62.
3. Sreerama N, Woody RW. Estimation of protein secondary structure from circular dichroism spectra: comparison of CONTIN, SELCON, and CDSSTR methods with an expanded reference set. Anal Biochem 2000;287(2):252-60.
4. Toumadje A, Alcorn SW, Johnson WC, Jr. Extending CD spectra of proteins to 168 nm improves the analysis for secondary structures. Anal Biochem 1992;200(2):321-31.
5. Bohm G, Muhr R, Jaenicke R. Quantitative analysis of protein far UV circular dichroism spectra by neural networks. Protein Eng 1992;5(3):191-5.
6. Louis-Jeune C, Andrade-Navarro MA, Perez-Iratxeta C. Prediction of protein secondary structure from circular dichroism using theoretically derived spectra. Proteins 2011;80(2):374-381.
7. Wiedemann C, Bellstedt P, Gorlach M. CAPITO--a web server-based analysis and plotting tool for circular dichroism data. Bioinformatics 2013;29(14):1750-7.
8. Micsonai A, Moussong E, Wien F, Boros E, Vadaszi H, Murvai N, Lee YH, Molnar T, Refregiers M, Goto Y and others. BeStSel: webserver for secondary structure and fold prediction for protein CD spectroscopy. Nucleic Acids Res 2022;50(W1):W90-W98.
9. Micsonai A, Wien F, Kernya L, Lee YH, Goto Y, Refregiers M, Kardos J. Accurate secondary structure prediction and fold recognition for circular dichroism spectroscopy. Proc Natl Acad Sci U S A 2015;112(24):E3095-103.
10. Muta H, Lee YH, Kardos J, Lin Y, Yagi H, Goto Y. Supersaturation-limited amyloid fibrillation of insulin revealed by ultrasonication. J Biol Chem 2014;289(26):18228-38.
11. Whitmore L, Woollett B, Miles AJ, Klose DP, Janes RW, Wallace BA. PCDDB: the Protein Circular Dichroism Data Bank, a repository for circular dichroism spectral and metadata. Nucleic Acids Res 2010;39(Database issue):D480-6.
